# Desmoplastic Fibroma of the Maxilla: A Report of a Rare Case

**DOI:** 10.7759/cureus.105354

**Published:** 2026-03-17

**Authors:** Marimuthu P, Keerthy Ajay Kumar

**Affiliations:** 1 Oral and Maxillofacial Surgery, Dr. Ziauddin Ahmed Dental College and Hospital, Aligarh Muslim University, Aligarh, IND; 2 Anatomy, All India Institute of Medical Sciences, Raebareli, Raebareli, IND

**Keywords:** benign aggressive tumor, bone tumor, desmoplastic fibroma, fibroblastic tumor, maxilla, maxillofacial pathology, odontogenic region

## Abstract

Desmoplastic fibroma (DF) is an uncommon benign tumor of bone that is notable for its locally destructive nature and frequent recurrence. The lesion originates within the osseous tissue and most often affects the mandible, pelvic girdle, and long bones; involvement of the maxilla is rare. An 18-year-old female patient presented with a slowly developing lesion in the right maxillary premolar area. Clinical examination supported by radiographic findings demonstrated an intraosseous pathology, and histopathological evaluation confirmed the diagnosis of desmoplastic fibroma. Although benign, this tumor exhibits aggressive local growth, highlighting the importance of prompt diagnosis and definitive surgical treatment, particularly in rare maxillary cases, to reduce the risk of functional impairment and esthetic deformity.

## Introduction

Desmoplastic fibroma (DF) is a rare benign bone tumor known for its locally aggressive behavior and infiltrative growth pattern. Histologically, it resembles desmoid tumors of the soft tissues. First described by Jaffe in 1958, DF is composed of dense collagenous stroma with spindle-shaped fibroblasts. Despite its aggressive local expansion, the tumor has minimal metastatic potential. The term desmoplastic is derived from the Greek word desmos, meaning ligament or band, reflecting the fibrous nature of the lesion [1].

DF constitutes less than 1% of all primary bone tumors and approximately 0.3% of benign osseous tumors [2]. It most frequently affects long bones such as the tibia, femur, and scapula. Within the craniofacial region, the mandible is the most commonly involved site, whereas maxillary involvement is notably rare [3]. Multifocal DF is extremely uncommon, with only isolated cases reported in long and pelvic bones [4]. Most patients are young, with approximately 92% of cases occurring in individuals younger than 30 years, and no definite gender predilection has been reported [5].

**Figure 1:**
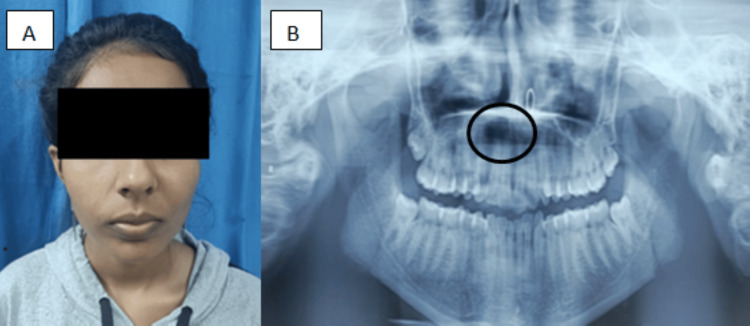
(A) Extraoral image of the patient and (B) preoperative panoramic view (A) Extraoral photograph showing mild facial asymmetry due to a swelling in the right midfacial region. (B) In the radiograph, the marked circle indicates unilocular radiolucency in relation to the right canine and premolar region.

Clinically, DF usually presents as a slow-growing, painless swelling. Radiographically, it appears as a well-defined radiolucency that may be unilocular or multilocular, often associated with cortical thinning, expansion, or perforation. Extension into adjacent soft tissues may occur, resulting in an appearance that can mimic malignancy [2]. Histopathologically, DF is characterized by spindle-shaped fibroblasts embedded in a dense collagenous stroma, with minimal cellular atypia and rare mitotic figures [1]. Because desmoplastic fibroma rarely involves the maxilla, documentation of such presentations is valuable for improving clinical recognition and diagnostic accuracy.

## Case presentation

An 18-year-old female patient presented to the Department of Oral and Maxillofacial Surgery at Dr. Ziauddin Ahmed Dental College and Hospital with a complaint of a painless swelling in the right maxillary region that had been gradually increasing in size over a period of six months. The swelling was not associated with difficulty in mastication, speech, or breathing. There was no history of trauma, nasal discharge, or sensory deficit. The patient’s medical, dental, and family histories were unremarkable.

Extraoral examination revealed a well-defined swelling measuring approximately 4 × 3 cm in the right midfacial region, resulting in mild facial asymmetry and partial obliteration of the nasolabial fold. The swelling extended from approximately 1 cm below the infraorbital rim inferiorly and laterally toward the cheek. The overlying skin appeared normal, and no sinus discharge or regional lymphadenopathy was noted. On palpation, the lesion was soft in consistency and non-tender (Figure 1A).

**Figure 2:**
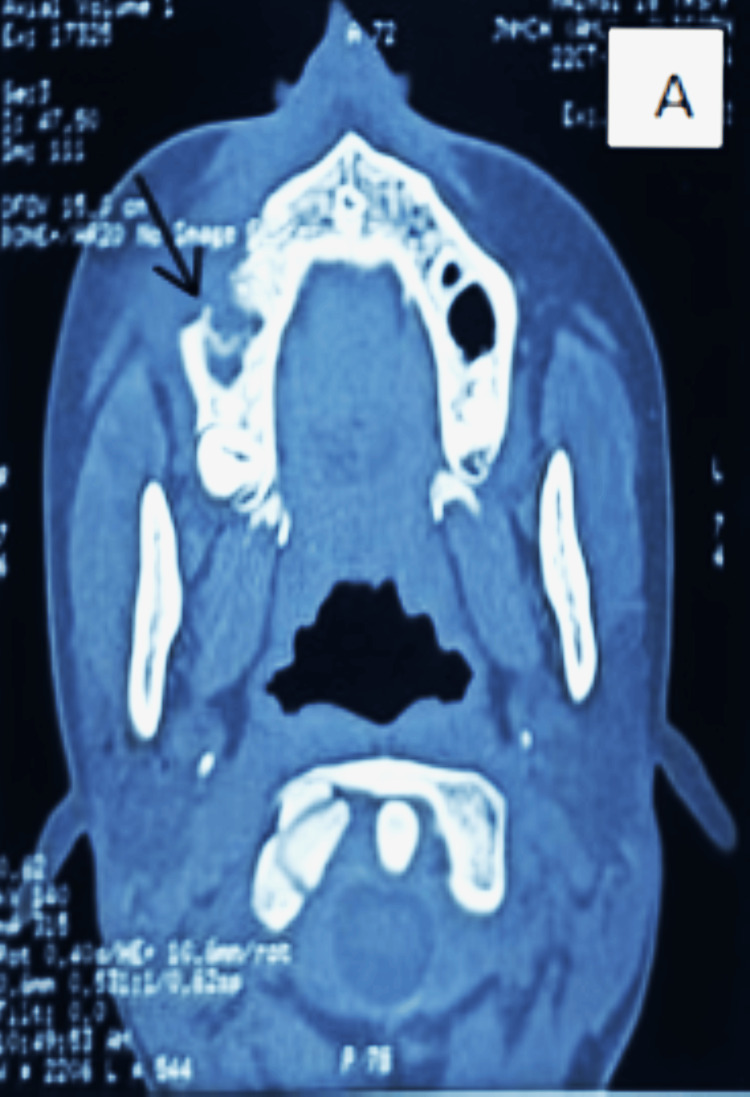
(A) Preoperative contrast-enhanced computed tomography (axial view) (A) Marked arrow indicates a radiolucent lesion with cortical resorption in the right maxilla.

Intraorally, mild obliteration of the buccal vestibule was noted in the right maxillary premolar region. Teeth associated with the lesion responded positively to cold and electric pulp vitality tests. Panoramic radiography revealed a well-circumscribed unilocular radiolucency in the right maxillary canine-premolar area (Figure 1B). Computed tomography demonstrated a radiolucent lesion with cortical resorption of the right maxillary bone (Figure 2A). Routine hematological investigations were within normal limits, and aspiration of the lesion yielded negative results (Table 1).

**Table 1:** Baseline laboratory investigations Table 1 shows all blood investigations within normal ranges.

Laboratory parameters	Patient value	Normal range
Hemoglobin (g/dL)	11.6	12-16
Total leukocyte count (×10⁹/L)	4.5	4-11
Platelet count (×10⁹/L)	250	150-400
Erythrocyte sedimentation rate (mm/hour)	6	0-20
Serum calcium (mg/dL)	9	8.6-10.2
Serum phosphorus (mg/dL)	3.6	2.5-4.5
Alkaline phosphatase (IU/L)	110	44-147

Surgical excision of the lesion was performed intraorally via crevicular incision from the distal to the right lateral canine to the mesial to the right first molar region under general anesthesia. The excised specimen was submitted for histopathological examination. Postoperative care instructions were provided, and the patient was monitored through regular follow-up visits. Regular irrigation was done with diluted 10% Betadine with normal saline to maintain oral hygiene.

**Figure 3:**
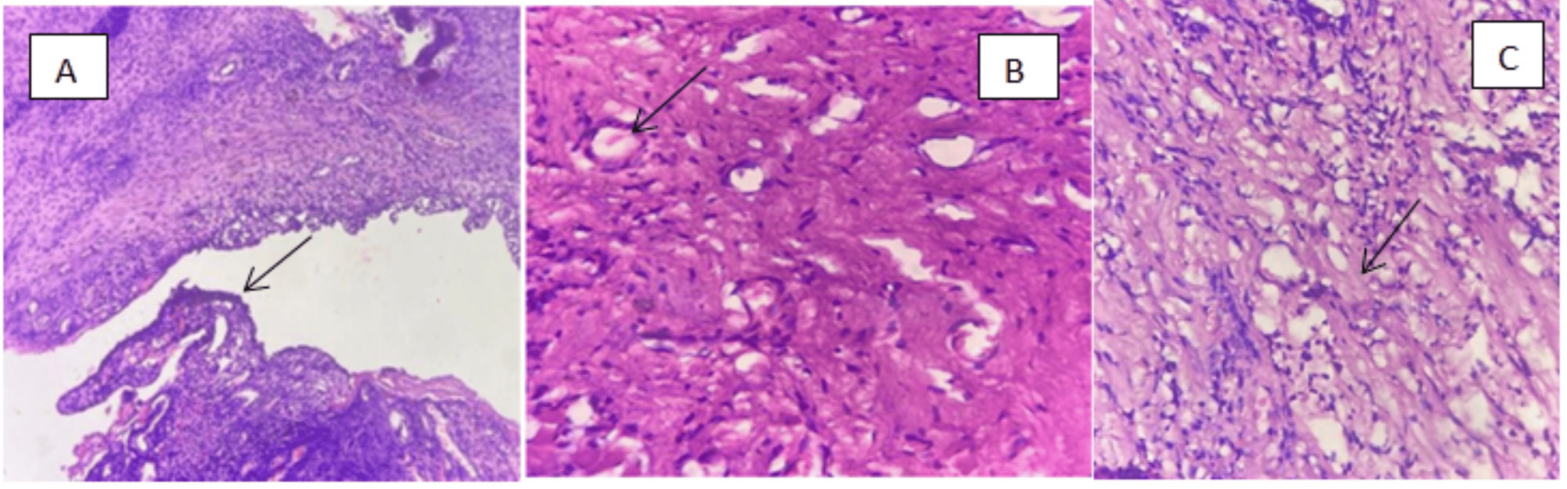
Histopathology image: (A) H&E, 10×; (B) H&E, 40×; and (C) H&E, 40× (A) Marked arrow indicates pseudostratified ciliated columnar epithelium. (B) Marked arrow indicates areas of hemorrhage. (C) Marked arrow indicates fibrocollagenous stroma. H&E: hematoxylin and eosin

Histopathological examination revealed variably cellular fibrocollagenous tissue in a focal fascicular pattern with pseudostratified columnar epithelium, along with dense mixed inflammatory infiltrate and areas of hemorrhage, supporting the diagnosis of desmoplastic fibroma (Figure 3A-3C).

The postoperative course was uneventful. No evidence of recurrence was observed during follow-up evaluations conducted at one month, three months, six months, and one year.

## Discussion

Desmoplastic fibroma is an uncommon intraosseous tumor with locally aggressive behavior and a high propensity for recurrence. Although benign, it can cause extensive bone destruction and functional impairment if inadequately treated. Its occurrence in the maxilla is exceptionally rare [3].

DF predominantly affects young individuals and shows no consistent gender predilection [5]. In the jaws, the mandible is more commonly involved than the maxilla, particularly the posterior body, angle, and ramus [3]. Anterior maxillary involvement, as seen in the present case, is unusual and adds to the rarity of this presentation.

Radiographically, desmoplastic fibroma may appear as an expansile radiolucent lesion with cortical thinning or perforation, which can resemble other benign jaw pathologies. Central giant cell granuloma typically shows multilocular radiolucency with numerous multinucleated giant cells on microscopy. Aneurysmal bone cyst is characterized by rapidly enlarging lesions containing blood-filled spaces. Odontogenic keratocyst presents as a cystic lesion lined by keratinized epithelium. In contrast, fibrous dysplasia usually exhibits a ground-glass radiographic pattern with immature woven bone in a fibrous stroma [2]. Confirmation of desmoplastic fibroma requires histopathological evaluation.

Histological differentiation of desmoplastic fibroma from other fibrous lesions is important. Myofibroma shows myofibroblastic proliferation with a characteristic biphasic pattern, unlike the dense collagen bundles seen in desmoplastic fibroma. Odontogenic fibroma contains strands of odontogenic epithelium, which are absent in desmoplastic fibroma. Cemento-ossifying fibroma demonstrates mineralized deposits such as bone or cementum-like material, features not present in desmoplastic fibroma. In contrast, fibrous dysplasia is characterized by irregular woven bone within fibrous stroma. The absence of odontogenic epithelium, mineralized tissue, significant atypia, and increased mitotic activity supports the diagnosis of desmoplastic fibroma [6].

Wide surgical excision is generally considered the most effective treatment for desmoplastic fibroma due to its locally aggressive and infiltrative nature. Treatment by curettage alone may lead to a higher chance of recurrence. Removing the lesion completely with adequate margins helps reduce this risk. Early diagnosis and appropriate surgical management contribute to better clinical outcomes. Regular postoperative evaluation is important to monitor healing and detect any recurrence. Therefore, long-term follow-up is recommended for proper patient management [4].

## Conclusions

Desmoplastic fibroma is an uncommon benign bone tumor characterized by locally aggressive growth and potential recurrence when inadequately treated. Its occurrence in the maxilla is rare and may complicate diagnosis because the clinical and radiographic features can resemble other jaw lesions. In the present case, correlation of imaging findings with histopathological evaluation was crucial for establishing the diagnosis. Surgical removal with adequate margins was performed to minimize recurrence. Prompt recognition and appropriate management are important to preserve function and facial form while ensuring favorable long-term outcomes.
